# Report of a Foreign Body in the Œsophagus, and Its Transit through the Alimentary Canal; Quick and Safe Recovery

**Published:** 1859-04

**Authors:** J. W. Redden

**Affiliations:** Wapella, De Witt County, Illinois.


*Report of a Foreign Body in the Œsophagus, and its Transit through the Alimentary Canal; Quick and Safe Recovery.* By J. W. Redden, M. D., of Wapella, De Witt County, Illinois. Read before the De Witt County Medical Society, on Monday, July 5th, 1858.

Mr. Chairman and Gentlemen,—I propose offering a brief report of a case, which to me seems quite interesting and rather anomalous; and, in doing so, I trust you may be amply repaid for the time and attention it may occupy. It relates to the enlodgement of a foreign body in the œsophagus, and its transit through the alimentary canal.

Of all the varieties of disease which are chronicled in the nosology of the medical science, perhaps there are none which are attended with such immediate and alarming symptoms, and which call forth so extensively the sympathy and anxiety of bystanders, as *foreign bodies* in the air passages and alimentary canal. It is a time when parents and friends are agitated, confused and boisterous. It is then the physician's skill and ability are tested. It is then judgment and discrimination are valuable; for he is called upon to act promptly, boldly, correctly. All eyes are centered upon him. Hopes and fears commingle. He must calm their commotion, quiet their fears, and exert his professional ingenuity in his unceasing efforts to rescue a fellow-being from impending dissolution.

On Monday morning, May 17, 1858, about ten o'clock, A. M., was called, with Dr. Wright, to see an infant. The messenger was in great haste, and stated that the child had just swallowed a piece of glass and was nearly suffocated. In a few minutes we reached the house, being but a short distance from our office. Found that the babe had been quite asphyxiated, but had just become relieved from the intense paroxysm. It was a girl ten and a half months old.

The mother had left the babe playing with its sister, and in a few moments returned to find her child in an alarming condition. Respiration slow and very laborious; child restless and much agitated; deep and dark venous cogestion of the neck and face; in fact animation was nearly suspended. It rolled to and fro, gagged, and made some efforts to vomit, when instantly a favorable change occurred. The paroxysm abated, its face assumed its natural hue, she became calm and nursed readily. We ascertained from the little girl that her sister had swallowed a piece of glass. From her description, we supposed it a long, narrow piece of window glass. A careful examination of the throat revealed nothing unusual. We gave it as our opinion that the foreign body had passed into the stomach. The mother was assured that for the present she need have no fears, and we advised her to watch the evacuations, for in all probability she would find, in a few days, the glass had passed impacted in the fæces. At the time, her health was very good, the bowels regular, and the fæces of natural consistence. Hence, we advised no medication, but requested the mother to be watchful, and if any alarming symptoms arose, straightway to make it known.

Now, some may suppose that here is a proper occasion to interpose an objection, and suggest that this was the proper time for the administration of a prompt emetic, or brisk cathartic, and also contend that such a plan of treatment would have been far more appropriate. We opine not; for our judgment assured us that this would have directly interfered with the acts of kind nature—the invaluable friend of medical art, without which man's skill is limited. Therefore, for a moment, let us pause and settle the objection.

If an active emetic had been given, true, it might have dislodged the *body* from the stomach, but in the efforts to throw it off, would it not in all probability have produced serious laceration, or even fatal impaction? We think so.

But what of the cathartic? Surely, it would have diminished the consistence of the fæces, increased the peristaltic motion of the intestines, and very likely have lacerated their coats, ending in perforation and death. Then, could any thing have been gained by this plan? Certainly not. Could any other course more appropriate be suggested? No.

Here, we will remark, that the management of the case was satisfactory and gratifying, as its termination fully proves; for, on Wednesday afternoon following, about four o'clock, the parents had the delight to find that the foreign body had passed, encased with fæces, being about fifty-six hours since it was swallowed.

In a short time we received the specimen. Upon examination and measurement, we find it a concavo-convex, angular and very irregular piece of glass—perhaps a fragment of a tumbler. It is about $\frac{3}{4}$ of an inch in length, varying from 1-16 to 5-16 of an inch in width, and from 1-16 to $\frac{1}{4}$ of an inch in thickness.

*Theory.*—The babe, while having the glass in its mouth, no doubt made an effort at deglutition, at which time the glass passed through the pharynx into the œsophagus, where we think it became entangled. The body was liable to have become impacted in the pharynx, or especially at the entrance or exit of the œsophagus. I am rather inclined to think, however, that it became entangled at the aperture or entrance of the œsophagus; for, had it lodged in the pharynx, it is very plausible to suppose that the efforts and struggles of the child would have expelled it. And had it passed the entrance or constricted portion of the œsophagus, it would, quite likely, have passed through the œsophagus without impediment.

But having passed through the pharynx, it came to the mouth of the œsophagus; and in consequence of this canal being flattened, narrow and constricted here, from the larynx projecting backward, it is possible—yea, highly probable—that at this point the foreign body became impacted; and from the distension and irritation of the parts, spasm of the glottis supervened, impeding respiration, retarding circulation, and, as the symptoms indicated, would soon have resulted in true asphyxia. For if a foreign body becomes impacted in the œsophagus, it produces a sense of choking and fits of suffocative cough, and if unrelieved, this may soon lead to suffocation by spasm of the glottis. But through the struggles of the child and its efforts to vomit, the spasm became relieved, and the foreign body, by its own gravitation, passed into the stomach. We think it must have passed base foremost, through the intestinal tube, guarded by its fæcal case. Be this as it may, it is an ugly, uneven and dangerous piece of glass, and would naturally lead one to apprehend the supervention of alarming symptoms. During its transit through the intestinal canal, no one could give other than a guarded and doubtful prognosis; for how easily might it have been detained impacted and lead to active peritonitis, and, perchance, fatal perforation. But no symptoms, either alarming or apprehensive, resulted.

The child seemed calm, playful, jovial; evinced no pain, fever, or peevishness. For a few hours, however, following the passage of the glass, it was rather cross and irritable, resulting, no doubt, from the irritation during its transit through the rectum.

The child is now one year old; is active, robust, and has every indication of uninterrupted health.

What seems most remarkable in this case is, the age of the child, the size of the glass and the facility with which it passed.

This is one of those emphatic cases, which so clearly and forcibly verifies the ancient maxim, "Vis Medicatrix Naturæ." It should also teach all physicians ever to evince proper discrimination, to judge when, and when not, to act; for it should always be remembered that meddlesome interference is a dangerous thing. And never can we be too forcibly impressed, in all our acts and observations through life, that Art is only the handmaid of Nature.

